# Atypical *BRAF* and *NRAS* Mutations in Mucosal Melanoma

**DOI:** 10.3390/cancers11081133

**Published:** 2019-08-08

**Authors:** Nicolas Dumaz, Fanélie Jouenne, Julie Delyon, Samia Mourah, Armand Bensussan, Céleste Lebbé

**Affiliations:** 1 INSERM, U976, Team 1, Human Immunology Pathophysiology & Immunotherapy (HIPI), F-75010 Paris, France; 2 Institut de Recherche Saint Louis (IRSL), Université de Paris, F-75010 Paris, France; 3 Département de Pharmacogénomique, Hôpital Saint Louis, AP-HP, F-75010 Paris, France; 4 Département de Dermatologie, Hôpital Saint Louis, AP-HP, F-75010 Paris, France

**Keywords:** mucosal melanoma, *BRAF*, *NRAS*, mutations, MAPK, targeted therapies

## Abstract

Primary mucosal melanomas represent a minority of melanomas, but have a significantly worse prognosis than cutaneous melanomas. A better characterization of the molecular pathogenesis of this melanoma subtype could help us understand the risk factors associated with the development of mucosal melanomas and highlight therapeutic targets. Because the Mitogen-Activated Protein Kinase (MAPK) pathway plays such a significant role in melanoma development, we explore v-raf murine sarcoma viral oncogene homolog B (*BRAF)* and neuroblastoma RAS viral oncogene homolog (*NRAS)* mutations in mucosal melanoma and compare them to the mutation profiles in cutaneous melanoma and other tumors with *BRAF* and *NRAS* mutations. We show that in addition to being less frequent, *BRAF* and *NRAS* mutations are different in mucosal melanoma compared to cutaneous melanomas. Strikingly, the *BRAF* and *NRAS* mutation profiles in mucosal melanoma are closer to those found in cancers such as lung cancer, suggesting that mutations in mucosal melanoma could be linked to some genotoxic agents that remain to be identified. We also show that the atypical *BRAF* and *NRAS* mutations found in mucosal melanomas have particular effects on protein activities, which could be essential for the transformation of mucosal melanocytes.

## 1. Introduction

Although the majority of melanomas have a cutaneous origin, approximately 5% arise from the eye (ocular melanoma) or mucosa (mucosal melanoma). Mucosal melanomas originate from melanocytes located in the nasopharyngeal, genitourinary, anorectal, and esophageal mucosal membranes. Although primary mucosal melanomas are rare, they are associated with a significantly worse prognosis compared to other melanoma subtypes, with the lowest five-year survival rate [1,2]. This poor prognosis can be attributed to a delay in diagnosis of the disease due to a lack of symptoms during early stages of the disease and to its occurrence in occult anatomic locations that are generally not amenable to self-examination. It is notable that mucosal melanomas occur more frequently among Asian populations than among Caucasian populations [3,4]. For recent reviews on mucosal melanomas focused on clinical and pathological data, which are outside the scope of this article, see [5]. Knowledge of mucosal melanoma pathogenesis and risk factors is insufficient when compared with cutaneous and ocular melanoma, where ultraviolet radiation that causes a predominant CG → TA nucleotide transition signature has been established as the major risk factor [6]. A better characterization of the molecular pathogenesis of mucosal melanoma could help us understand the risk factors associated with the development of mucosal melanomas. The rarity of mucosal melanomas has also hindered the genomics-era precision medicine advances that have helped patients with cutaneous melanomas. Indeed, nearly half of cutaneous melanomas harbor activating *BRAF* V600E/K mutations, and therefore, can be treated with BRAF kinase inhibitors in combination with an MEK inhibitor [7]. However, *BRAF* V600E/K mutations are less common in mucosal melanoma, rendering them less amenable to BRAF/MEK inhibitor therapies. Targeted therapy against v-kit Hardy-Zuckerman 4 feline sarcoma viral oncogene homolog (*KIT)* activating mutations, seen in around 10–22% of mucosal melanomas [8], has been tested in clinical trials but results have been underwhelming. Treatment with KIT inhibitors resulted in a trend toward improved response in melanoma patients, with response rates of approximately 20%. Despite the clinical benefit achieved with KIT inhibition in select patients with melanoma harboring *KIT* mutations, most patients ultimately experience disease progression [9,10,11,12,13,14]. Immunotherapy has recently emerged as a promising treatment modality for cutaneous melanomas. However, only 20–30% of patients with mucosal melanoma respond to anti-PD-1 immunotherapy [15], and in addition, the median progression-free survival after anti-PD-1 immunotherapy in these patients is reportedly short, at approximately 4 months [16]. The combination of anti-PD1 with anti-CTLA4 seems to have greater efficacy than either agent alone, but the efficacy of the combined immunotherapies is still lower in mucosal than cutaneous melanomas [16]. A better characterization of the molecular pathogenesis of mucosal melanoma could offer new hope for the development of more effective systemic therapies. Several whole-exome sequencing studies on mucosal melanomas have been published, but they do not agree on the most frequent genetic alterations in mucosal melanomas, probably due to differences in patient ethnic origins between the cohorts analyzed. Indeed, mucosal melanomas seem to present recurrent mutations in *BRAF, CTNNB1, DMXL2, GNAQ, GNA11, KIT, NF1, RAS, SF3B1,* and *SPRED1*, among others [17,18,19,20,21,22,23]. Here, seeking to further characterize the molecular pathogenesis of mucosal melanoma, we explored *NRAS* and *BRAF* mutations in mucosal melanoma and compared them to the mutation profiles in cutaneous melanoma and other tumors with mutations in *NRAS* and *BRAF*. We discovered that there is very limited data comparing the type of *NRAS* and *BRAF* mutations in mucosal and cutaneous melanomas. However, we were able to show that in addition to being less frequent, *NRAF* and *BRAF* mutations are different in mucosal melanoma compared to cutaneous melanomas, with mucosal melanoma mutations being strikingly closer to the type of mutations found in cancers such as lung cancers, raising fundamental questions about their etiology.

## 2. MAPK Pathway

**Figure 1:**
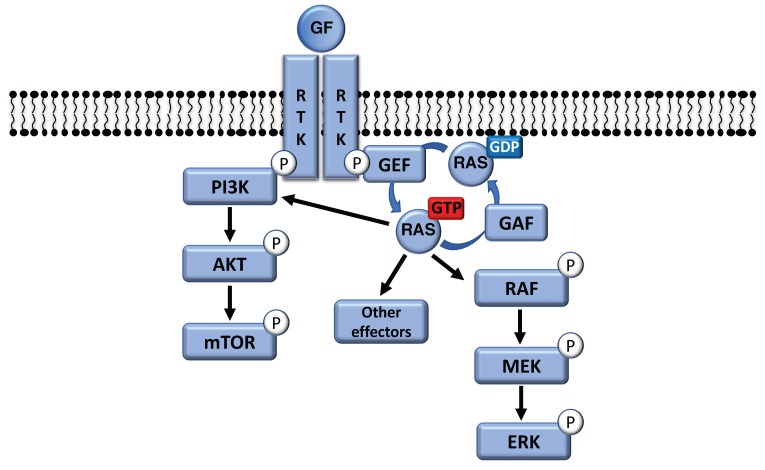
The PI3K/AKT/mTOR and the RAS/RAF/MEK/MAPK pathways. GAF: GTPase activating protein; GEF: guanine nucleotide exchange factor; GF: growth factor; and RTK: receptor tyrosine kinase.

NRAS and BRAF both play a part in the mitogen-activated protein kinase (MAPK) pathway, which significantly contributes to melanoma development. In physiological conditions, the MAPK pathway is activated by growth factors binding to their surface receptor tyrosine kinase (RTK), and the signal is transmitted through the small GTPase RAS [24]. There are three closely related isoforms, HRAS, KRAS, and NRAS, which are expressed in all cells and have overlapping but distinctive functions. RAS proteins are 21kDa molecular switches that cycle between the inactive GDP-bound conformation and the active GTP-bound conformation (Figure 1) [25].

Guanine nucleotide exchange factors (GEFs) activate RAS by catalyzing the release of GDP, facilitating GTP binding, which induces a conformational change in the RAS revealing an effector binding site. RAS has slow intrinsic GTPase activity, which is accelerated by GTPase activating proteins (GAPs) binding to the GTP-bound Ras to allow rapid RAS inactivation [26]. Active RAS proteins will recruit effectors to membranes resulting in their activation by conformational change, phosphorylation, or interaction with a cofactor or substrate, leading to signal transduction [27]. Over 20 Ras effectors have been identified, although most work has focused on the serine/threonine kinases of the RAF family and the lipid kinase of the PI3K (PtdIns-3 kinase) family, which regulate cell proliferation and survival respectively (Figure 1). There are three RAF isoforms: ARAF, BRAF, and CRAF (also known as RAF1), which activate MAP kinase kinase (MEK1/2), which in turn activate the MAP kinases (ERK1/2). Activated ERK promotes cell proliferation and survival by phosphorylating multiple substrates both in the cytosol and in the nucleus [28,29,30]. Whilst the MAPK pathway is activated, in melanocytes, by growth factors such as SCF (Stem Cell Factor), EGF (Epidermal Growth Factor), FGF (Fibroblast Growth Factor), or HGF (Hepatocyte Growth Factor), this pathway is very often constitutively activated in melanoma due to the presence of activating mutations of *NRAS* and *BRAF*, and inactivating mutations of the GAP *NF1*. Mutations in *NRAS*, *BRAF*, and to a lesser extent *NF1*, are mutually exclusive because their oncogenic activity is linked to stimulation of the MAPK pathway [31,32]. We focused our work on *NRAS* and *BRAF* because there is sufficient genomic data available in mucosal melanoma.

## 3. NRAS

**Figure 2:**
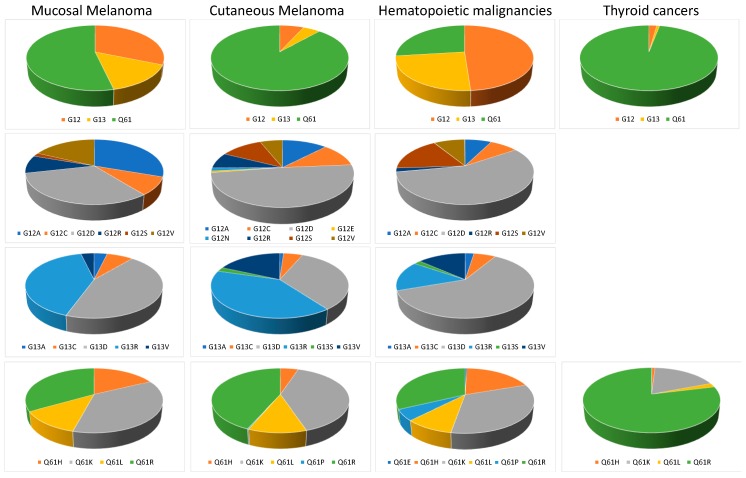
Oncogenic mutations of NRAS on glycine 12 (G12), 13 (G13), and glutamine 61 (Q61) collated from the literature (mucosal melanoma; Table S1) or from COSMIC (cutaneous melanoma, hematopoietic malignancies and thyroid cancers).

An intriguing observation from the early days of RAS research is that different types of cancer appear to be coupled to a mutation of a particular RAS isoform. For example, *KRAS*, which is the most frequently mutated isoform in cancer, is mutated in pancreatic, colorectal, and lung adenocarcinoma. In contrast, *NRAS* mutations are mainly found in melanoma, hematopoietic, and lymphoid tissue malignancies, and to a lesser extend thyroid tumors. *HRAS* mutations are rare but found mainly in head and neck squamous cell carcinoma [33]. Analyses of codon mutation frequencies reveal that each isoform has a distinctive codon mutation signature. *KRAS* mutations occur mainly at codon 12 (83%), whereas *NRAS* tumors mainly harbor mutations at codon 61 (63%). *HRAS* displays an approximately 40%/30%/30% split between mutations at codons 61, 12, and 13, respectively [34]. To better understand the molecular pathogenesis of mucosal melanoma we compiled *NRAS* mutations in 1387 mucosal melanoma from 36 publications and added the data from 67 mucosal melanomas from our own research center (Table S1). The analyzed mucosal melanomas were located on the head and neck (46%), genital area and urinary tract (26%), anus and rectum (13%), and digestive tract (4%). The location of the rest (11%) was not specified. We focused our analysis on *NRAS* mutations located on codons G12, G13, and Q61 because RAS mutations outside these codons are not yet clearly established as driver mutations. The data showed that *NRAS* mutations were present in 12% (179/1454) of mucosal melanoma, and 54% (96/179) were located on Q61, 31% (56/179) on G12, and 15% (27/179) on G13 (Figure 2).

**Figure 3:**
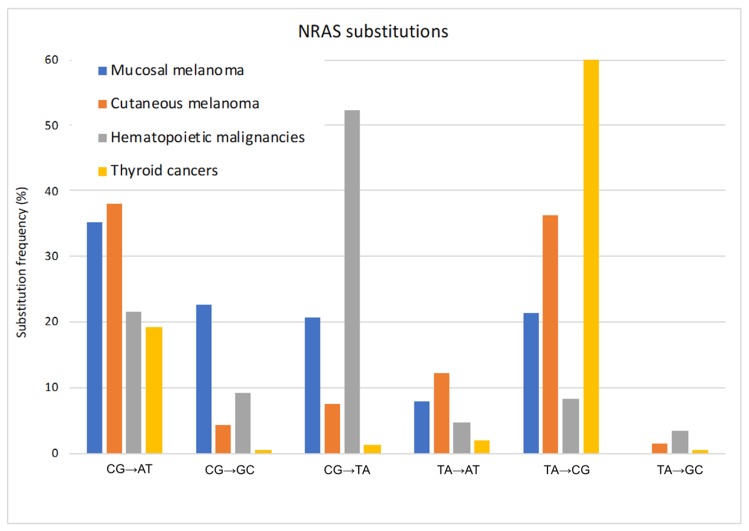
Proportion of somatic base changes in NRAS.

We compared those data with *NRAS* mutations in cutaneous melanomas, hematopoietic and lymphoid tissue malignancies, and thyroid cancers from The Catalog of Somatic Mutations in Cancer (cancer.sanger.ac.uk) [35]. We found that *NRAS* exhibits distinctive codon mutations and amino acid substitutions in melanoma compared to hematopoietic and lymphoid tissue malignancies and thyroid cancers, as could be expected. Thyroid cancers showed almost exclusively Q61 mutations (97%), and hematopoietic tumors were strongly associated with mutations at G12 (49%) and G13 (24%) (Figure 2). Surprisingly however, the comparison also showed a noticeable difference between cutaneous and mucosal melanomas regarding the location of *NRAS* mutations. Although the most frequent types of *NRAS* mutations are located in codon 61 for both cutaneous melanoma (88%) and mucosal melanoma (54%), mutations at codons 12 and 13 occurred more frequently in mucosal melanomas (46%) than cutaneous melanomas (12%). In all malignancies, the most commonly observed *NRAS* codon 61 mutations are the Q61R (CAA/CGA) and Q61K (CAA/AAA) changes (Figure 2). Mutations at codon 13 were mainly G13R (GGT/GCT) and G13D (GGT/GAT), but with a prevalence of G13R in cutaneous melanoma, and of G13D in mucosal melanoma and hematopoietic malignancies. (Figure 2) Alterations of codon 12 were predominantly G12D in cutaneous melanomas and hematopoietic malignancies, and a combination of G12A and G12D in mucosal melanomas (Figure 2). These data highlight mutational biases, which could be due to differences in exposure to mutagens. This was exemplified when we examined the preferred single-base substitutions, which revealed a final level of difference among the mutation profile in the different malignancies. Hematopoietic tumors presented mainly CG → TA mutations, thyroid cancers TA → CG mutations, and cutaneous melanomas CG → AT and TA → CG, whereas mucosal melanomas presented the different substitutions more uniformly (Figure 3).

Strikingly, although ultraviolet radiation causes a predominant CG → TA nucleotide transition signature, this substitution is rare in cutaneous melanoma. Although *NRAS* mutations in cutaneous melanoma do not present the typical UV signature, Q61 mutations have previously been linked to UV. The *NRAS* wild-type codon 61 CAA contains a TT pyrimidine doublet in the noncoding strand and is a site for the formation of potential mutagenic cyclobutane dimers and (6–4) pyrimidine photoproducts, as demonstrated by UV irradiation experiments in vitro [36]. Furthermore, the third nucleotide in the noncoding triplet could also be converted by UV-light induction to an 8-oxo-deoxyguanosine, which is known to mispair with adenosine [37]. A mutagenic effect of UV irradiation could be demonstrated after UV irradiation of a cloned human *NRAS* proto-oncogene in vitro and subsequent transfection, leading to the codon 61 Q61R (CAA/CGA) and Q61K (CAA/AAA) changes that are identical to those found in cutaneous melanoma samples [38]. Furthermore, UV photoproducts in UV irradiated human skin fibroblasts were mapped at a high frequency to codon 61 of the transcribed strand of all three RAS genes, but rather rarely to codons 12 and 13 [36]. Finally, UV-induced skin tumors in a C3H mouse contained mutations preferentially in the *NRAS* oncogene and frequently opposite or adjacent to dipyrimidine sites [39]. *NRAS* mutations on Q61 found in cutaneous melanoma are hence consistent with known mechanisms for UV induction. The lower frequency of Q61 mutations in mucosal melanoma compared to cutaneous melanoma suggests that the *NRAS* mutations in the former are not linked to UV irradiation. With its higher frequency of mutations at G12 and G13, causes of mucosal melanoma could have been the same as hematopoietic malignancies. However, the *NRAS* mutation spectra are different, suggesting that the etiologies of are different. This is interesting because mutations in hematopoietic malignancies are not associated with a specific mutagen, but rather due to a combination of proliferation-dependent mutation incorporation, spontaneous deamination of cytosine, and defects in repair processes. These data suggest that mutations in *NRAS* in mucosal melanomas are neither UV-induced nor spontaneous, but may be due to genotoxic agents, which remain to be identified.

## 4. BRAF

**Figure 4:**
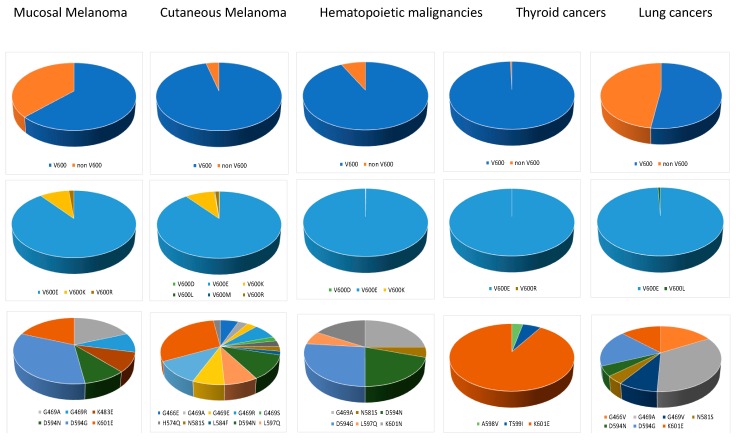
Oncogenic mutations of *BRAF* on valine 600 (V600) or outside valine 600 (non-V600). For non-V600 mutations, we only show point mutations representing more than 2% of non-V600 alterations in the different malignancies.

*BRAF* mutations are present in approximately 8% of human tumors, but with huge variation in frequency depending on the malignancy. *BRAF* is commonly mutated in melanomas (50%), papillary thyroid cancers (45%), hairy cell leukemias (100%), and idiopathic disorder Langerhans cell histiocytosis (50–60%), and less frequently in colorectal cancers (10%), lung adenocarcinomas (10%), and hematopoietic and lymphoid tissue malignancies (8%) [40]. *BRAF* missense mutations in tumors encompass 115 of the 766 BRAF amino acids, but most of the mutations occur in the activation loop (A-loop) near V600, or in the GSGSFG phosphate-binding loop (P-loop) at residues 464–469 [40]. The most frequent mutation is a substitution T to A in exon 15, resulting in an amino acid change in the activation segment of BRAF at codon 600 from valine (V) to glutamic acid (E). In wild-type BRAF, reversible phosphorylation of T599 and S602 in the A-loop regulates its interaction with the P-loop to control BRAF kinase activity [41]. Accordingly, *BRAF* mutations in either the A-loop or the P-loop are supposed to mimic T599 and S602 phosphorylation, irreversibly disrupting the A-loop–P-loop interaction and inducing a several-fold kinase hyperactivation [41]. To gain insight into the molecular pathogenesis of mucosal melanoma, we compiled *BRAF* mutations in 1312 mucosal melanoma from 33 publications and added the data from 27 mucosal melanomas from our own research center (Table S2). The data showed that *BRAF* mutations were present in 8% (107/1339) of mucosal melanoma, 63% (67/107) were located on the V600 codon, and 37% (40/107) on another codon (Figure 4). We compared those data with *BRAF* mutations in cutaneous melanomas, hematopoietic and lymphoid tissue malignancies, thyroid cancers, and lung adenocarcinoma from The Catalog of Somatic Mutations in Cancer. The comparison shows that not only are *BRAF* mutations less frequent in mucosal melanoma, but there is also a noticeable difference regarding the location of *BRAF* mutations between both melanoma subtypes. We found that whereas cutaneous melanomas present a vast majority of V600 mutation (more than 90%), like hematopoietic and lymphoid tissue malignancies and thyroid cancers, mucosal melanomas are characterized by a high prevalence of non-V600 mutations (37%), as in lung adenocarcinomas (48%; 259/543) (Figure 4).

In all malignancies, V600E is the most commonly observed *BRAF* codon 600 mutation (at least 89%), followed in melanomas by V600K (9%) and V600R (1–2%) (Figure 4). We then compared the non-V600 mutations focusing on point mutations representing more than 2% of non-V600 alterations in the different malignancies. Looking at the location of the mutated codon, we noticed that mucosal melanomas showed mutations on D594 (40%), G469 (24%), and K601 (16%), which is similar to the mutation spectrum in hematopoietic and lymphoid tissue malignancies (40%, 22%, and 18%, respectively), but different from the one in cutaneous melanoma, which presents mutations on K601 (30%), L597 (24%), D594 (17%), and G469 (15%) (Figure 4). As seen with *NRAS* mutations, *BRAF* data highlight mutational biases, which could be due to differences in exposure to mutagens. This was exemplified when we examined the preferred amino acid substitutions, which reveal differences among the mutation profiles for the different malignancies. In thyroid cancers, non-V600 mutations are almost exclusively K601E (91%) mutations. In cutaneous melanomas, apart from the prevalence of the K601E (30%) mutation, a wide range of non-V600 mutations is presented. This is different from the mucosal melanomas, which show a prevalence of D594G (33%), G469A (19%), and K601E (19%), similar to the mutation spectrum in lung adenocarcinomas (19%, 34%, and 13%, respectively). By contrast, hematopoietic tumors present D594G (27%), G469A (25%), D594N (20%), and K601N (17%) (Figure 4). Therefore, the *BRAF* mutation spectrum in mucosal melanomas is different from the spectrum seen in cutaneous melanomas (UV-induced) or in hematopoietic malignancies (spontaneous), but is closely related to the mutation spectrum seen in lung cancers where mutations are often associated to the genotoxic effects of cigarette smoking. Similarly to *NRAS, BRAF* mutations suggest the existence of genotoxic agents in mucosal melanoma, which remain to be identified.

## 5. Discussion

The analysis of somatic mutations in tumors provides insight into the mutational processes that have shaped the cancer genome. Examining the preferred single-base substitutions collated from *NRAS* and *BRAF* in mucosal melanoma revealed a different mutation profile than cutaneous melanoma, suggesting different etiologies for both malignancies. Although these data suggest the existence of a genotoxic agent responsible for the specific mutation spectra in mucosal melanoma, biological reasons could also contribute to the hotspot mutation preferences. Indeed, the specific *NRAS* and *BRAF* mutants found in mucosal melanoma could be linked to their particular effects on protein activity during mucosal melanocyte transformation, adding an additional layer of complexity.

### 5.1. Q61 Versus G12 RAS Mutants

Historically, it was assumed that all oncogenic mutations on amino acid G12, G13, and Q61 generated equivalent effects on protein activity. However, more than 60% of the mutations for each isoform are accounted for by only 3 of the 18 potential mutations across the codon, suggesting that not all RAS mutants are equal. In support of this idea, HRAS G12V exhibits weaker GTPase activity and stronger binding to GTP than HRAS G12D, and it is also more potent in cell culture-based transformation assays [42,43]. In colorectal and lung cancers, KRAS G12V mutations have been associated with a worse prognosis than KRAS G12D mutations, raising the possibility that particular amino acid substitutions might dictate specific transforming characteristics of oncogenic RAS alleles. Moreover, the Q61L, Q61V, and Q61K mutant variants transform NIH 3T3 cells nearly 300-fold and 1000-fold more efficiently than the Q61G and Q61E mutants, respectively [44]. There is also increasing evidence that mutations at each of the three missense-mutation hotspots (G12, G13, and Q61) cause distinct structural and biochemical defects as well as cell-specific differences [44,45,46,47]. In agreement with this hypothesis, it was reported that, in the p16INK4a-deficient mouse melanoma model, the frequency of metastatic melanoma initiation by NRAS Q61R was increased more than 20-fold, compared with NRAS G12D [45]. The mechanistic basis for the enhanced oncogenic activities of NRAS Q61 mutants in melanoma remains to be clearly established. Nonetheless, there is evidence that the Q61 mutants, through a malfunction of the allosteric switch, have a very strong oncogenic effect in tumors where the RAF-MEK-ERK pathway is primarily involved in promoting transformation [34]. This would explain the high frequency of NRAS Q61 and BRAF V600 mutants in cutaneous melanoma where the MAPK pathway is playing such a significant role. In contrast, the prevalence of G12 and G13 NRAS mutants in mucosal melanoma raises the intriguing possibility that the RAF-MEK–ERK pathway could have a lesser role in the transformation of mucosal melanocytes. In a mouse lung tumorigenesis comparing Q61L/R to G12V/D mutants, it was shown that Q61L/R is more potent than G12V/D at activating KRAS (assessed by KRAS-GTP) and driving the MAPK pathway. Q61L/R mutations were the only KRAS oncogenes that induced detectable p16 expression in primary lung fibroblasts and resulted in more potent growth arrest than G12V/D mutations. However, Q61L/R mutations were rarely detected in lung tumors developing after the administration of urethane, raising the interesting prospect that the more potent Q61L/R mutations were selected against in favor of the weaker G12V/D mutations, which may evade a growth-arrest response [48]. A similar mechanism could explain the prevalence of weaker G12/G13 mutations in mucosal melanomas.

### 5.2. V600 Versus Non-V600 BRAF Mutants

Although most studies on the role of *BRAF* in melanoma have focused on the *BRAF* V600E mutation, several other mutations in the *BRAF* gene have been identified, and the biochemistry of the various altered *BRAF* proteins has been shown to vary substantially [49]. This is interesting because, although *BRAF* mutations are rare in mucosal melanoma, they are characterized by a higher prevalence of non-V600 mutations than in cutaneous melanoma. Many of these mutants show a lower BRAF kinase activity toward MEK in vitro than that of the V600E mutant, explaining why these mutants are often classified as “low activity” or “impaired activity” mutants (for example, G469A and D594G are found frequently in mucosal melanomas). However, in vivo, these mutants are able to promote MEK phosphorylation in a CRAF-dependent manner by directly binding to and activating CRAF [50]. In a genetically engineered mouse model, conditional melanocyte-specific expression of either BRAF D594A or KRAS G12D was insufficient to induce nevi or melanomas. However, co-expression of both mutant proteins promoted cellular dimerization of the catalytically inert BRAF D594A with the catalytically competent CRAF, inducing melanoma [51]. Under these circumstances, a feedback loop from BRAF to itself is interrupted, and the non-phosphorylated BRAF protein cooperated with RAS and CRAF to induce MEK phosphorylation, presumably mimicking the effects of BRAF kinase inhibitors. These data strongly indicate that kinase-impaired *BRAF* mutations are oncogenic drivers, but require activated RAS and CRAF to activate downstream signaling. However, so far, only one mucosal melanoma carrying both mutants (*BRAF* D594E + *NRAS* G13R) has been described [52]. Nonetheless, the wild-type RAS has been shown to mediate the same effect when kicked into the active GTP-bound states by upstream signals. Alterations of upstream effectors of RAS, such as *NF1* or *SPRED1*, in mucosal melanomas could play this role, but there is currently not enough extensive genomic data on mucosal melanoma to confirm this hypothesis [22,53]. Beyond the BRAF dimerization-induced activation through activated RAS, cell-based studies also showed that certain oncogenic BRAF mutants, such as L597V or G466E, promote spontaneous BRAF dimerization and activation by forming homodimers in the absence of RAS-GTP [54,55]. The exact mechanism by which these mutations induce dimerization of the kinase is not well understood. Because the impaired activity mutants are frequently found in mucosal melanomas, their homodimerization or heterodimerization with CRAF should play a major role in the transformation of mucosal melanocytes. The fact that their oncogenic and transactivation potential depends on an intact dimerization interface and on RAS activity has an important implication for targeted therapy (see below). In addition to driving the MAPK pathway, it was also shown that the BRAF impaired activity D594A mutant can promote aneuploidy. In a conditional knock-in mouse model, the BRAF D594A mutant does not drive tumor development per se, but it is able to induce aneuploidy in murine splenocytes and mouse embryonic fibroblasts and contributes to immortalization through the propagation of aneuploid cells. The emergence of an aneuploid phenotype is dependent on CRAF but independent of MEK-ERK, whereas the growth of aneuploid cells depends on both [56]. Therefore, the BRAF D594A mutant not only drives aneuploidy in a MEK-ERK independent manner, but also activates MEK-ERK to overcome the growth-inhibitory effect of aneuploidy, and hence, facilitates the emergence of aneuploid cells with a growth advantage. These results provide a link between impaired activity BRAF mutants and chromosomal instability, which could play a role in the development of mucosal melanomas. In accordance with this hypothesis, mucosal melanomas have a genetic landscape characterized by structural rearrangements and amplifications [57].

## 6. Conclusions

Many variants have been described in the context of mucosal melanoma, but besides KIT mutations, there is still no clear consensus on the most frequent driver mutations. We catalogued alterations of the *NRAS* and *BRAF* genes in mucosal melanoma and showed that they represent a significant portion of mutations (20%) in these tumors. We also demonstrated that mutations in *NRAS* and *BRAF* in mucosal melanoma are different from the ones found in cutaneous melanoma, suggesting the existence of genotoxic agents in mucosal melanoma that remain to be identified. Irritants and carcinogenic substances, such as tobacco smoke and formaldehyde, could be a risk factor even though the evidence for this is very low. For oral mucosal melanomas, cigarette smoking has been suggested as risk factor because it has been demonstrated that pigmented oral lesions are more prevalent among smokers [58]. Exposure to formaldehyde has also been suggested to be a risk factor for sinonasal mucosal melanomas, since cases have been reported among workers subject to industrial or professional exposure to this substance [59]. Moreover, the *BRAF* mutation spectrum in mucosal melanoma is closely related to the mutation spectrum seen in lung cancers where mutations are often associated with the genotoxic effects of cigarette smoking. Epidemiological studies are now required to evaluate these risk factors in the development of mucosal melanomas.

The peculiar *NRAS* and *BRAF* mutation spectra in mucosal melanomas could also be linked to different oncogenic potencies as well as distinct cell-specific functional consequences [60]. It is thus possible that the prevalence of specific *NRAS* mutations is related, in some measure, to the activation of other RAS effector pathways to complement RAF activation for mucosal melanoma development. The specific *NRAS* and *BRAF* mutations could also impair the magnitude of oncogenic signaling to, for example, prevent senescence. In accordance with this hypothesis, it is interesting to note that, in two studies of genital nevi, the *BRAF* V600E mutation was found in 76% (26/34) and the *NRAS* Q61K in 3% (1/34) of nevi and no G12/G13 *NRAS* or non-V600E *BRAF* mutations were detected [61,62]; this is in clear contrast to the mutations found in genital melanomas. These results suggest that there may be a narrow window of oncogenic RAS-BRAF signaling necessary for transformation of mucosal melanocytes requiring “less active” NRAS or BRAF mutants.

The presence of *NRAS* and *BRAF* mutations in mucosal melanomas raises the question of their therapeutic potential for this particularly deadly form of melanoma, which has not yet fully benefited from genomics-era advances in precision oncology. Although the first generation of BRAF inhibitors aimed at the V600E mutant are not suitable for treating the atypical *BRAF* mutations, second-generation BRAF inhibitors and CRAF inhibitors could be used. In melanoma cells harboring the low-activity *BRAF* mutations (D594G or G469E), targeting CRAF with either sorafenib or small interfering RNAs decreases ERK phosphorylation and induces apoptosis [63]. The type IIa BRAF inhibitor PLX7904 and its optimized analogue PLX8394 have been shown to inhibit signaling driven by V600 and non-V600 mutants in lung adenocarcinomas, where 48% of *BRAF* mutant tumors have non-V600 mutations [64]. ARAF might also be a therapeutic target in *NRAS*-mutated melanoma, as it was recently established that ARAF could mediate MAPK pathway activation under specific conditions in melanoma. Moreover, rare activating mutations of ARAF have been identified in melanoma associated with an *NRAS* mutation, reinforcing the potential role of ARAF in *NRAS*-induced melanoma [65]. Finally, mucosal melanomas mutated on *BRAF* and *NRAS* may also be sensitive to MEK inhibitors as well as MEK inhibitor-based combinations. The next generation of MEK inhibitors (Trametinib, Binimetinib, and Selumetinib) have shown promising clinical efficacy even in *NRAS*-mutant melanoma [66,67].

In summary, the findings of the present study refine our understanding of the role of *BRAF* and *NRAS* mutations in mucosal melanomas and could pave the way for therapeutic intervention. The clarification of the contribution of the atypical *NRAS* and *BRAF* mutations to mucosal melanoma pathogenesis will however require the development of suitable cellular and animal models for this melanoma subtype.

## Supplementary Materials

The following are available online at https://www.mdpi.com/2072-6694/11/8/1133/s1, Table S1: *NRAS* mutations in mucosal melanoma, Table S2: *BRAF* mutations in mucosal melanoma.
